# Genome-wide identification and expression analysis of the JMJ-C gene family in melon (*Cucumis melo* L.) reveals their potential role in fruit development

**DOI:** 10.1186/s12864-023-09868-3

**Published:** 2023-12-13

**Authors:** Wuyun Jin, Wei Yan, Ming Ma, Agula Hasi, Gen Che

**Affiliations:** Key Laboratory of Herbage & Endemic Crop Biology, Ministry of Education, School of Life Sciences, Inner Mongolia University, Hohhot, 010070 China

**Keywords:** Jumonji-C (JMJ-C) family, Melon, Sequence analyses, Expression profile, Gene localization, Fruit development

## Abstract

**Background:**

Proteins with the jumonji (JMJ)-C domain belong to the histone demethylase family and contribute to reverse histone methylation. Although JMJ-C family genes have an essential role in regulating plant growth and development, the characterization of the JMJ-C family genes in melon has not been uncovered.

**Results:**

In this study, a total of 17 JMJ-C proteins were identified in melon (*Cucumis melo* L.). *CmJMJ*s were categorized into five subfamilies based on the specific conserved domain: KDM4/JHDM3, KDM5/JARID1, JMJD6, KDM3/JHDM2, and JMJ-C domain-only. The chromosome localization analyses showed that 17 *CmJMJs* were distributed on nine chromosomes. Cis-acting element analyses of the 17 *CmJMJ* genes showed numerous hormone, light, and stress response elements distributed in the promoter region. Covariance analysis revealed one pair of replicated fragments (*CmJMJ3a* and *CmJMJ3b*) in 17 *CmJMJ* genes. We investigated the expression profile of 17 *CmJMJ* genes in different lateral organs and four developmental stages of fruit by RNA-seq transcriptome analysis and RT-qPCR. The results revealed that most *CmJMJ* genes were prominently expressed in female flowers, ovaries, and developing fruits, suggesting their active role in melon fruit development. Subcellular localization showed that the fruit-related *CmJMJ5a* protein is specifically localized in the cell nucleus.

**Conclusions:**

This study provides a comprehensive understanding of the gene structure, classification, and evolution of *JMJ-C* in melon and supports the clarification of the *JMJ-C* functions in further research.

**Supplementary Information:**

The online version contains supplementary material available at 10.1186/s12864-023-09868-3.

## Background

Epigenetics denotes the enzymatically reversible modifications of corresponding gene expression without any alteration of the DNA sequences, which can be stably inherited by the progeny [1]. Epigenetic processes include DNA methylation, nucleosome remodeling, covalent modification of histones, and noncoding RNA regulation [2–6]. The epigenetic mechanisms of histone modification activate or inhibit transcription by regulating the chromatin state or directly recruiting specific effector proteins, which in turn modulate chromatin accessibility or the activity of the underlying DNA [7–9]. The homeostasis of histone methylation is dynamically regulated by histone methyltransferases (HMTs) and histone demethylases (HDMs), which add or remove methyl groups on specific residues to make transcription factors facilitate or prevent access to genomic DNA [10, 11]. Jumonji-C (JMJ-C) family proteins belong to histone demethylases, which remove methylation marks by hydroxylation relying on Fe(II) and *a*-ketoglutarate (α-KG) with broader specificity [12]. JMJ-C protein function is involved in the epigenetic regulation of tumorigenesis and pathogen infection in animals [13], leaf senescence [14], floral transition [15, 16], the control of seed germination [17], the regulation of shoot regeneration [18], and rhythm-related processes in plants [19]. Genes encoding JMJ-C have been characterized in several plant species, including *Arabidopsis thaliana* [20], *Zea mays* [21], *Citrus sinensis* [22], *Oryza sativa* [23], *Gossypium hirsutum* [24], *Solanum lycopersicum* [25] and *Jatropha curcas* [26]. In Arabidopsis, 21 JMJ-C proteins were identified and categorized into different subfamilies based on sequence similarity and catalytic specificity, including KDM4/JHDM3, KDM5/JARID1, JMJD6, KDM3/JHDM2, and JMJ-C domain-only [20]. KDM5/JARID subfamily proteins can remove the methylation of H3K4me1/2/3 [27]. *AtJMJ16* gene negatively regulates leaf senescence by reducing the expression level of H3K4me3 and inhibiting the expression of the leaf maturation genes *WRKY53* and *SAG201* [28]. *AtJMJ15* and *AtJMJ18*, which also belong to the KDM5/JARID subfamily, both can stimulate flowering by decreasing the methylation level of H3K4 at the *FLC* locus and subsequently suppressing *FLC* expression, thereby promoting *FLOWERING LOCUS T* (*FT*) activity in the accompanying cells [29]. KDM4/JHDM3 subfamily genes eliminate the methylation of H3K9me2/3 and H3K36me2/3 [30], and KDM3/JHDM2 subfamily proteins have H3K9me1 and H3K9me2 demethylation activity [31]. JMJD6 subfamily can demethylate H3K27me2/3 [32], and JMJ-C domain-only subfamily proteins can remove the methylation of H3K27me3 [33].

JMJ-C proteins are involved in the expression of multiple genes, chromatin activity, and biological processes such as plant growth, metabolism and environmental responses. In Arabidopsis, *AtJMJ27* negatively regulates the expression of the flowering regulatory factor *CONSTANS* (*CO*) and positively regulates *FLOWERING LOCUS* C (*FLC*) gene activity, leading to delayed flowering time [34]. At a suitable temperature, *AtJMJ30* and *AtJJMJ32* jointly remove H3K27me2/3 from *FLC* to prevent a premature flowering phenotype [35]. *AtJMJ30* and *AtJJMJ32* are also involved in mediating the abscisic acid (ABA) response during root development at the seedling stage by removing H3K27me3 from *SnRK2.8* to activate its expression [36]. AtJMJ30 binds to *LBD16* and *LBD29* and promotes leaf callus transition by activating *LBD* expression [37]. AtJMJ27 is a histone H3K9 demethylase that directly associates with the drought stress positive regulators *GOLS2* and *RD20* to participate in drought stress [38]. In rice, *OsJMJ706* has H3K4me1/2/3 demethylase activity, can affect rice flower morphology development [39] and *OsJMJ703* participates in plant defense against drought stress [40]. In *Medicago truncatula*, *MtJMJC5* undergoes cold-dependent alternative splicing and may be involved in the response to freezing tolerance [41]. In higher plants, fruit development is a vital biological process, which is regulated by gene regulatory network and epigenetic modification. In tomato, *SlJMJ6* overexpression specifically demethylated H3K27 methylation and up-regulated the ripening-related genes, including *SlRIN*, *SlACO1*, *SlACS4*, *SlPL*, *SlTBG4* and *SlDML2*, and thus accelerated the fruit development [25]. Overexpression of *SlJMJ7* specifically demethylates the methylated H3K4me3 and inhibited the *SlDML2* expression, which resulted in the delayed fruit ripening [42]. In banana, *MaJMJ15* regulated the fruit maturation by removing H3K27me3 from their chromatin to activate the expression of several key RRGs [43].

Melon (*Cucumis melo* L.) is a widely cultivated horticultural crop with high nutritional and economic value. Melon fruits undergo dramatic changes in size, color, texture, nutrition, and aroma during development, and the fruit ripening has both climacteric and non-climacteric genotypes. Therefore, melon can be used as a good model plant to study the genetic mechanism of fruit development based on their diverse agronomic traits, such as sex determination, stress tolerance, fruit development and ripening processes. Although JMJ-C family proteins participate in varying aspects of plant growth in different species, their gene features and potential roles in melon have not been characterized. In this study, 17 *CmJMJ* genes were identified in melon. Then, we performed a comprehensive bioinformatic study of chromosomal location, phylogenetic relationships, genomic organization, conserved protein domains, cis-acting elements, gene duplication events, and synteny relationships. Additionally, the expression profiles of *CmJMJ*s were analyzed in different tissues and four stages of fruit development. This study provides a molecular basis for further functional research on the JMJ-C domain-containing proteins in melon.

## Results

### Identification of *JMJ-C* genes in *Cucumis melo* L.

**Fig. 1:**
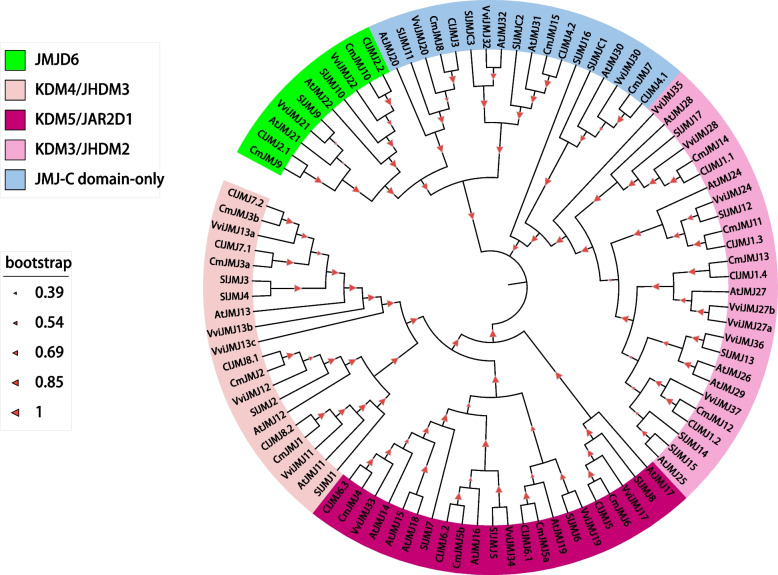
Phylogenetic relationship of JMJ-C family proteins in melon (*Cucumis melo*), tomato (*Solanum lycopersicum*), grape (*Vitis vinifera*), watermelon (*Citrullus lanatus*), and *Arabidopsis* (*Arabidopsis thaliana*). The phylogenetic tree was constructed by MUSCLE and Neighbor-Joining (NJ) method with 1000 bootstrap replicates in MEGA 7.0. JMJ-C proteins were clustered into five groups based on relative sequence homology. Blocks of subfamily KDM4/JHDM3, KDM5/JARID1, JMJD6, KDM3/JHDM2, and JMJ-C domain-only were displayed in different color, as shown in the upper left corner of the figure

We performed genome-wide identification of CmJMJ proteins by a combinatorial approach of the domain characteristics, CDD, and InterPro database. A total of 17 *JMJ-C* genes were identified and then designated *CmJMJ1* to *CmJMJ15* (*CmJMJ3a* to *CmJMJ3b* and *CmJMJ5a* to *CmJMJ5b*) based on homology between the melon and *Arabidopsis* genes (Fig. 1)*.* The basic information of *CmJMJs*, gene ID, molecular weight (Mw), isoelectric point (pI) and protein length are listed in Table 1. The protein length of CmJMJ showed various differences, which varied from 413 aa (*CmJMJ7*) to 1817 aa (*CmJMJ6*); the corresponding coding sequence length of the *CmJMJ* genes varied from 1242 bp (*CmJMJ7*) to 5454 bp (*CmJMJ6*); the molecular weights ranged from 46.68 kDa (*CmJMJ7*) to 208.73 kDa (*CmJMJ6*), and the predicted pI was between a minimum of 4.33 (*CmJMJ8*) and a maximum of 8.91 (*CmJMJ10*) (Table 1). The prediction of subcellular localization found that 17 melon *JMJ-C* gene family members were located in the nucleus, which was consistent with the occurrence of silencing redundant parts of the genome by histone demethylation to ensure the structural and functional integrity of the genome. The number of *JMJ-C* genes tends to be conserved in different species, with 21, 21, 20 and 20 *JMJ-C* genes in *Arabidopsis thaliana* [20], *Vitis vinifera* [44], rice (ChromDB database) [45] and *Solanum lycopersicum* [25], respectively. The reduced *JMJ-C* gene number in melon implied that several essential *CmJMJ* genes may undertake a comprehensive role in regulating plant growth during melon evolution.

**Table 1:** Chromosomal location of melon *CmJMJs* and physicochemical properties of proteins

Gene name	Gene ID	Chromosome	Location	CDS length/bp	Protein			
					AA	Mw (kDa)	pI	Subcellular localization
*CmJMJ1*	MELO3C019813.2	3	23,489,140–23497463 (-)	4848	1615	180.47	6.74	Nuclear
*CmJMJ2*	MELO3C002327.2	12	24,822,711–24,830,280 (-)	2562	853	93.82	6.22	Nuclear
*CmJMJ3a*	MELO3C003435.2	4	1,165,795–1,173,643 ( +)	1923	640	72.92	7.97	Nuclear
*CmJMJ3b*	MELO3C013826.2	6	33,793,389–33,804,248 (-)	2679	892	100.43	7.69	Nuclear
*CmJMJ4*	MELO3C021809.2	12	17,928,201–17933964 ( +)	3081	1026	116.30	5.86	Nuclear
*CmJMJ5a*	MELO3C008714.2	5	17,778,859–17,787,934 ( +)	3705	1234	138.28	7.35	Nuclear
*CmJMJ5b*	MELO3C012748.2	1	24,084,739–24089250 ( +)	2493	830	93.72	6.63	Nuclear
*CmJMJ6*	MELO3C005670.2	9	23,335,384–23,355,501 (-)	5454	1817	208.73	6.82	Nuclear
*CmJMJ7*	MELO3C015209.2	2	5,984,904–5989035 ( +)	1242	413	46.68	5	Nuclear
*CmJMJ8*	MELO3C026280.2	2	26,273,023–26277717 (-)	1365	454	52.18	4.33	Nuclear
*CmJMJ9*	MELO3C011038.2	3	29,182,217–29,192,951 (-)	2910	969	111.01	5.12	Nuclear
*CmJMJ10*	MELO3C009577.2	4	31,073,510–31075890 ( +)	1662	553	62.98	8.91	Nuclear
*CmJMJ11*	MELO3C004232.2	5	25,411,787–25,417,256 ( +)	2730	909	103.88	7.13	Nuclear
*CmJMJ12*	MELO3C025053.2	9	15,494,523–15,505,749 ( +)	3075	1024	116.20	6.91	Nuclear
*CmJMJ13*	MELO3C006019.2	6	605,711–619023 ( +)	2946	981	111.29	7.65	Nuclear
*CmJMJ14*	MELO3C007332.2	8	2,239,225–2,245,012 (-)	3018	1005	114.29	7.77	Nuclear
*CmJMJ15*	MELO3C015807.2	1	30,446,344–30468380 (-)	1797	598	67.49	5.99	Nuclear

### Phylogenetic Analysis of the *JMJ-C* genes

**Fig. 2:**
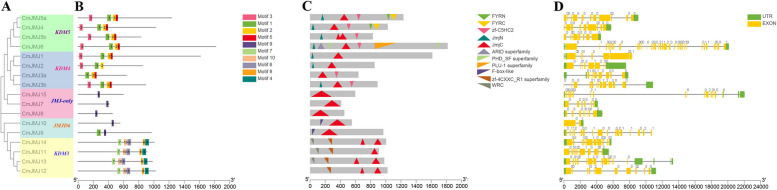
Comprehensive sequence analyses of the melon *CmJMJ* genes. **A** Phylogenetic relationship of CmJMJ proteins. The phylogenetic tree was performed using Neighbor-Joining (NJ) method and CmJMJ proteins were clustered into five groups. **B** Motif analysis. Ten motifs in CmJMJ proteins are indicated by different colored squares. **C** Schematic structure of conserved domains. The figure displays the position and size of the different conserved domain, which is indicated by colored triangle. **D** Gene structure analyses of the *CmJMJ* family genes. Green and yellow boxes represent UTR and exons, respectively. Black line represents the intron

To explore the evolutionary relationship of *CmJMJ* genes, we constructed a phylogenetic tree using 96 JMJ-C protein sequences referenced from *Cucumis melo*, *Vitis vinifera* (21), *Arabidopsis thaliana* (21), *Solanum lycopersicum* (20), and *Citrullus lanatus* (17) (Fig. 1). According to the JMJ-C subfamilies in *Arabidopsis*, the phylogenetic tree was branched into five groups, KDM4/JHDM3, KDM5/JARID1, JMJD6, KDM3/JHDM2, and JMJ-C domain-only, by blasting the sequence similarities and common conserved domains. Among them, KDM4/JHDM3, KDM5/JARID1 and KDM3/JHDM2 are the three largest subfamilies, and JMJD6 subfamily has a minimum of members (Fig. 1). In melon, the member of subfamilies showed that there are four gene members in each of KDM4/JHDM3, KDM5/JARID1 and KDM3/JHDM2 subfamilies, while the JMJD6 subfamily has two members (Fig. 2A).

### Conserved motifs and gene structure analysis of *CmJMJ-C* genes

We analyzed the conserved motif and gene structure of all 17 CmJMJs and identified 10 motifs in melon (Fig. 2B). KDM3/JHDM2 subfamily genes have motif4, motif6, motif7, motif8, and motif10, which is unique in the KDM3/JHDM2 family (Fig. 2B). In the KDM4/JHDM3 and KDM5/JARID1 subfamilies, most CmJMJs contain motif1, motif2, motif3 and motif5, and align in the order of 3-1-2-5 (Fig. 2B). The JMJ-only subfamily only contains motif9, and the JMJD6 subfamily was equipped with motif9 and motif1 (Fig. 2B). Motifs in the KDM3/JHDM2 subfamily genes were aligned in the order of 7-10-6-8-4. The regular motif alignments in the different subfamilies indicated the sequence conservation within subfamilies and validated the reliability of the phylogenetic tree division. The conserved domain analyses showed that all melon JMJ-C proteins have JMJ-C domain, and some of KDM3/JHDM2 subfamily proteins have two JMJ-C domains (Fig. 2C). In KDM5/JARID1 and KDM4/JHDM3 subfamilies, the JMJ-N was always synchronized with JMJ-C domain (Fig. 2C). FYRC and FYRN domains appeared in the CmJMJ4 and CmJMJ5a proteins, and the longest protein CmJMJ6 had the most domains, containing ARID, PLU-1, and PHD (Fig. 2C). The gene structure analyses in *CmJMJs* exhibit tremendous variation in the exon number, which ranges from 2 to 35 (Fig. 2D). We assumed that the evolutionary diversification of JMJ-C family protein structures and functions may be conferred by the loss or gain of their conserved domains.

### Chromosome distribution and collinear analysis of* CmJMJ-C* genes

**Fig. 3:**
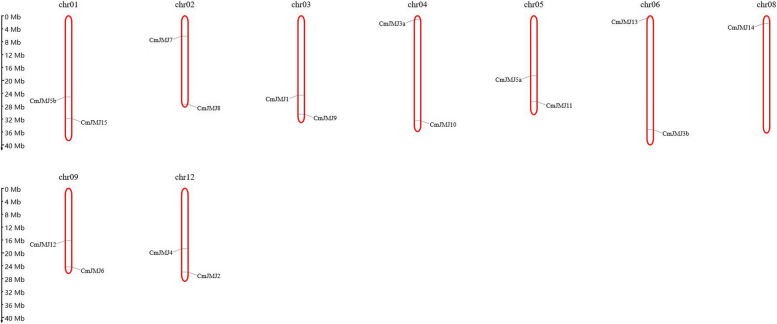
17 *CmJMJ* genes are mapped on the melon chromosomes. The nine chromosomes were indicated by numerals, and *CmJMJs* undistributed chromosomes were not shown. Black lines indicated the gene location

Chromosomal localization analysis revealed that *CmJMJ* genes were evenly distributed on nine chromosomes (Chr1, 2, 3, 4, 5, 6, 8, 9 and 12), which displayed two genes on each chromosome (Fig. 3). Nevertheless, only *CmJMJ14* is located on Chr08 (Fig. 3).

**Fig. 4:**
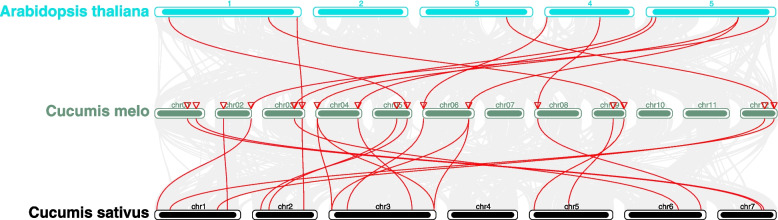
Collinear relationships of gene pairs from cucumber, melon and *Arabidopsis*. Red lines indicate the collinear *JMJ-C* gene pairs between different genomes

**Fig. 5:**
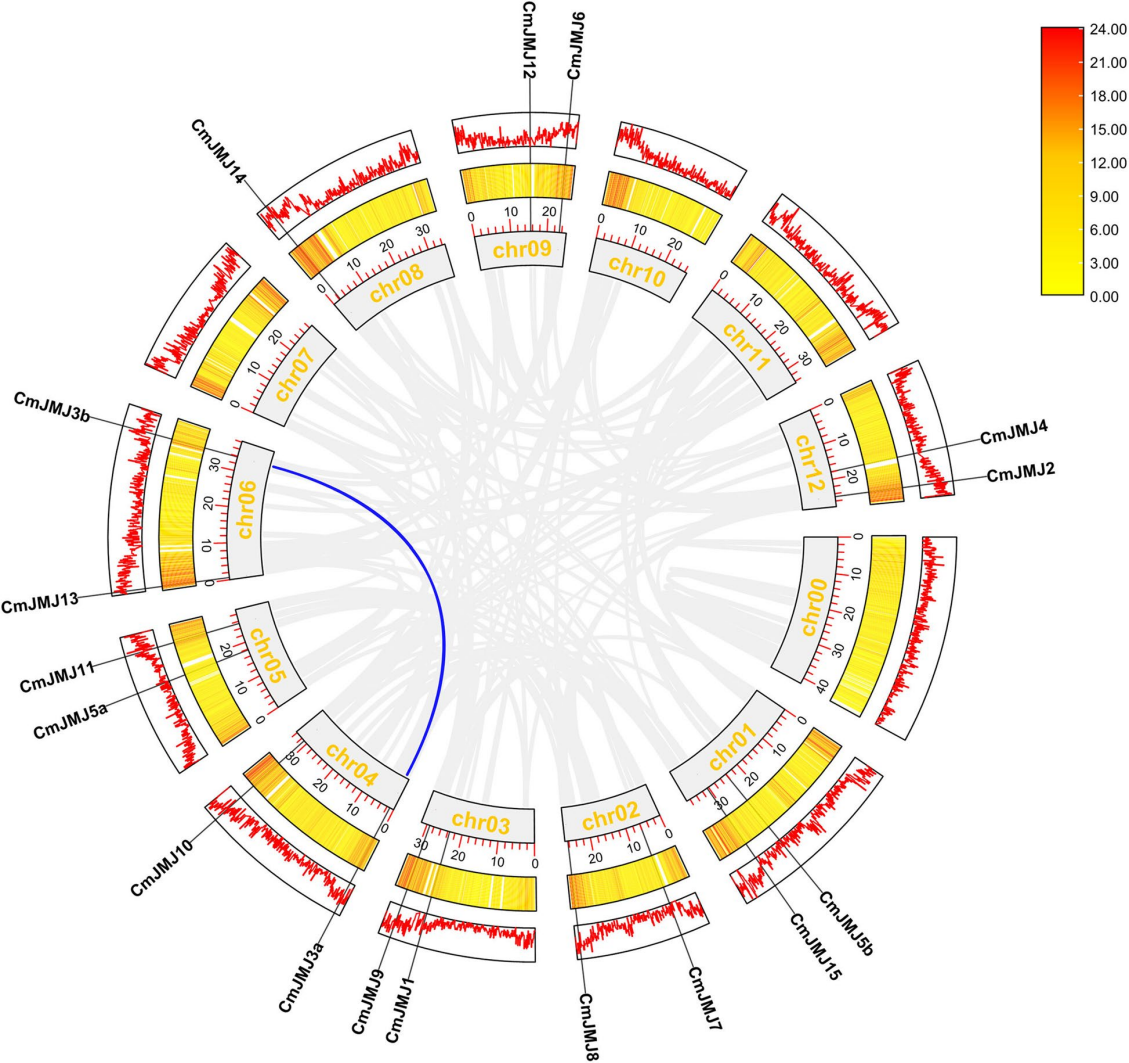
Synteny analysis of the melon genome and segmental duplications of *CmJMJs*. Gray lines denote the details of syntenic regions in the melon genome, and blue lines denote *CmJMJ* gene pairs with segmental duplication. The melon chromosomes arranged in the inner circle, and the scale numbers under the histograms indicate the chromosome size (Mb). The middle and outer circles indicate the gene density in the chromosomes

To reveal the evolutionary origin of the *CmJMJ* genes, a synteny analysis of interspecies collinearity between melon and two other species was conducted (Fig. 4). The gray lines show collinear blocks of different chromosomes, while the red line indicates the collinear *JMJ-C* gene pairs within melon and the two species (cucumber and *Arabidopsis*). We found 11 collinear gene pairs within the melon and *Arabidopsis* genomes when Arabidopsis Chr2 had no collinear gene with *CmJMJ*. A total of 17 pairs of putative *JMJ-C* genes were collinear between cucumber and melon. The increased number of orthologous genes in *Cucurbitaceae* may be attributed to their closer evolutionary relationship than Arabidopsis. Further covariance analysis found a pair of fragment replication (*CmJMJ3a* and *CmJMJ3b*) genes without tandem replication events within the *CmJMJ* family (Fig. 5). Combined with previous phylogenetic analyses, we found that both putative fragment replication genes belong to the same KDM3/JHDM2 subfamily and are on the same branch of the phylogenetic tree. We speculated that their replication events occurred late and functionally undifferentiated.

### Cis-element analysis of the promoter regions of the *CmJMJ* genes

**Fig. 6:**
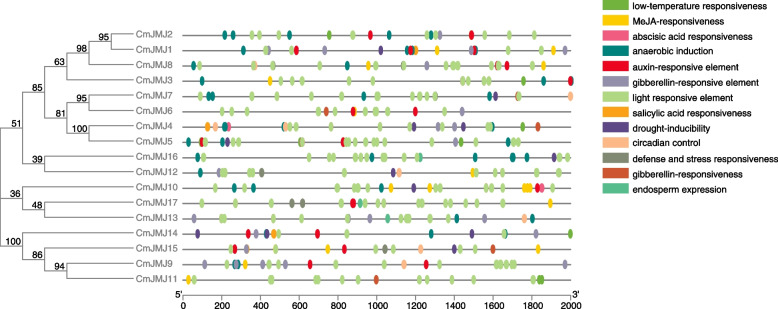
Cis-acting elements of the melon *CmJMJ* genes. The different colored ovals indicate the position and number of the cis-acting elements located in the 2000bp upstream promoter region

To further clarify the possible regulatory mechanisms of *CmJMJ* genes, we analyzed the cis-acting elements in the promoter regions (2000-bp upstream sequence from the translation start site) of *CmJMJ* genes by PlantCARE database (Fig. 6). We found the cis-acting elements involved in phytohormone (abscisic acid, gibberellin, MeJA, auxin and salicylic acid), light response, growth and development, and abiotic stress in *CmJMJ* genes. Light-responsive elements were found in all *CmJMJ*, and gibberellin-responsive, anaerobic induction, auxin-responsive element, MeJA-responsive and abscisic acid-responsive elements had the most common distribution. The hormone-related cis-acting elements were commonly presented in all subfamilies of *CmJMJ*: gibberellin-responsive elements were presented in the KDM3/JHDM2 and KDM4/JHDM3 subfamily genes, and MeJA-responsive elements were presented in all members of KDM5/JARID1. We also found that abiotic stress-related elements (low-temperature responsive, drought-inducible, defense and stress-responsive and anaerobic-inducible elements) were enriched in *CmJMJ*s. The endosperm expression element was found only in JMJ-only subfamily genes.

### Expression profiles of *CmJMJs* in melon

**Fig. 7:**
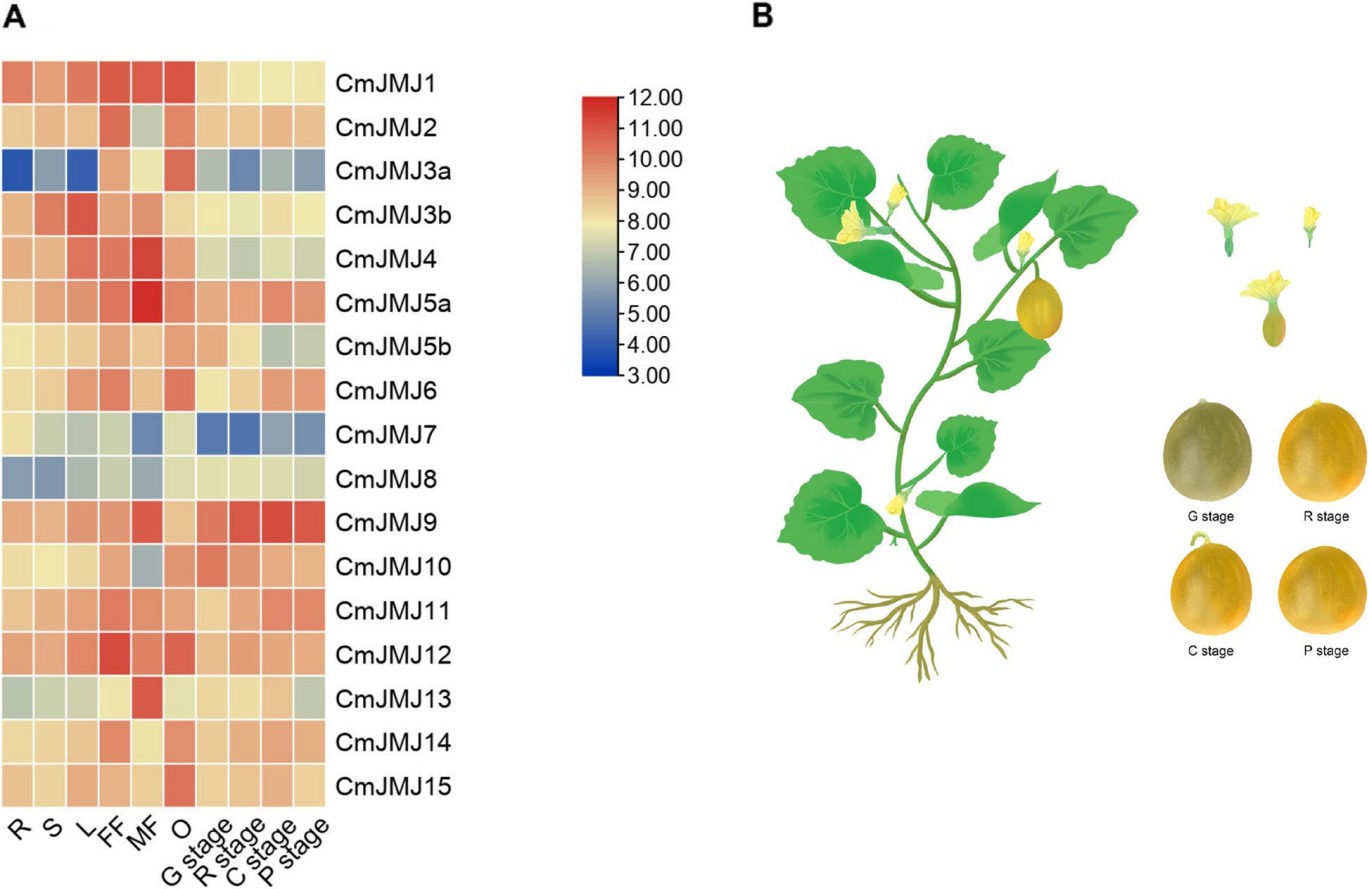
Expression profiles of *CmJMJ* genes in melon. **A** The heatmap and hierarchical cluster representing R (Root), S (Stem), L (Leaf), FF (Female Flower), MF (Male Flower), O (Ovary), and four different stages of fruit development: G (growing stage), R (ripening stage), C (climacteric stage), P (post-climacteric stage). **B** Schematic model of melon plant growth (left) and fruit development (right)

**Fig. 8:**
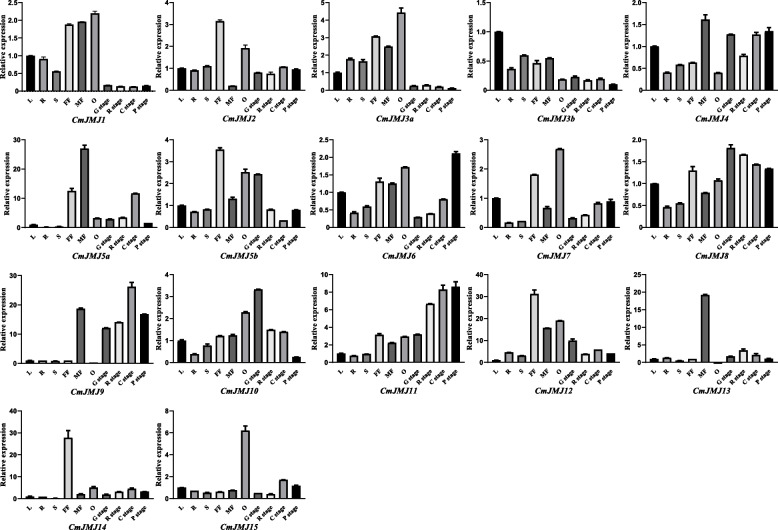
Relative expression of 17 *CmJMJ* genes in melon different tissues. L (leaf), R (root), S (stem), FF (female flower), MF (male flower), O (ovary), G stage (growing stage), R stage (ripening stage), C stage (climacteric stage), and P stage (post-climacteric stage). *GAPDH* was used as the reference gene, and gene expression in leaves was used as control “1”. Gene names are written under each histogram. The expression values were analyzed from three biological replicates and three technological replicates

We explored the expression profile of all 17 *CmJMJ* genes in melon different tissues, roots, stems, leaves, female flowers, male flowers, ovaries, and flesh of fruits at four developmental stages (G stage: growing stage, R stage: ripening stage, C stage: climacteric stage, P stage: postclimacteric stage), using the released melon transcriptomic data on NCBI (Fig. 7A). The transcript abundance of *CmJMJ* genes in different tissues fluctuated widely, which indicates their comprehensive role in different aspects of melon growth and development (Fig. 7B). High expression of *CmJMJs* was mostly accumulated in the female flower, male flower, and young ovary. Then, we performed RT-qPCR to further verify the expression of *CmJMJ* genes and found that most of the gene expression had a similar trend to the transcriptome analysis (Fig. 8). The expression levels of the *CmJMJ5a*, *CmJMJ6*, *CmJMJ9* and *CmJMJ11 *genes showed an increasing trend during the fruit ripening period from G to P (Fig. 7A), which may suggest their positive regulation in fruit ripening. *CmJMJ5b* and *CmJMJ10* showed a decreasing trend in fruit developmental stages, suggesting that they may be associated with the inhibition of fruit ripening. The RT-qPCR results were consistent with the RNA-seq analysis (Fig. 8). *CmJMJ1*, *CmJMJ3a*, *CmJMJ7*, and *CmJMJ15*, showed high expression in the young ovary and reduced expression in the later G R C P developmental stages of fruit, implying their active role in regulating early fruit growth instead of ripening. *CmJMJ2*, *CmJMJ11*, *CmJM12*, and *CmJMJ14* were highly accumulated in the female flower compared with other organs, while *CmJMJ5a* and *CmJMJ13* had the highest expression in the male flower, showing their different transcription level in floral sex differentiation.

**Fig. 9:**
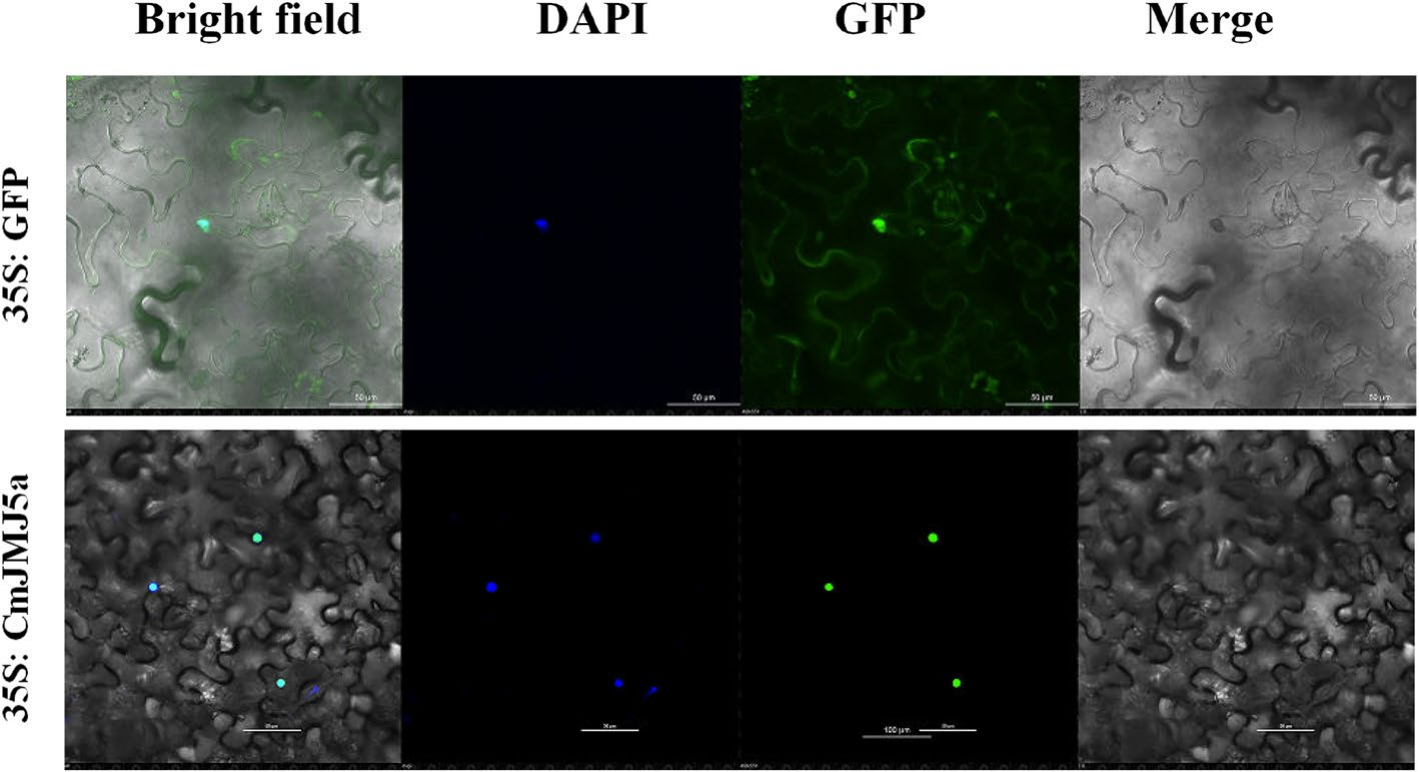
Subcellular localization of CmJMJ5a in tobacco leaves. CmJMJ5a is localized in the nucleus, based on visualization of green fluorescent protein (GFP) in tobacco (*Nicotiana benthamiana*) leaves via Agrobacterium-mediated transformation. Bars = 50 μm

To clarify the subcellular localization of CmJMJ protein, we cloned the *CmJMJ5a* coding sequence without stop codon fragment and constructed a gene-localizing vector. The young leaves of tobacco (*Nicotiana benthamiana*) were transformed by the positive control 35S:GFP and the recombinant vector 35S:CmJMJ5a-GFP. The results showed that CmJMJ5a-GFP fusion protein was only expressed in the nucleus (Fig. 9), confirming the predicted subcellular localization in the Table 1.

## Discussion

Plants have developed many biological capabilities to adapt the challenging environmental conditions during their evolution. Gene regulations and epigenetic modifications play vital roles in controlling plant biological processes, including root growth, gametophyte or embryo formation, floral organogenesis/senescence, and fruit development [46]. Histone methylation is an epigenetic modification that controls the dynamic balance by histone methyltransferases and demethylases [11]. JMJ-C domain–containing proteins are the largest family of histone demethylases in natural organisms. JMJ-C protein family have 21 members in model plant *Arabidopsis thaliana* [20], 20 members in rice [45], 20 members in *Solanum lycopersicum* [25], 21 members in *Vitis vinifera* [44], 20 members in *Citrus sinensis* [22], 18 members in *Citrus grandis* [47], 22 members in *Phyllostachys edulis* [48], and 28 JMJ-C members in *Rosa chinensis* [49]. We identified a total of 17 JMJ-C proteins in melon, showing a reduced member number compared to the other plants, which may because of the small genome size of melon and less gene duplication events during evolution. According to the sequence similarity and domain specification, the *CmJMJ* gene family in melon was classified into five subfamilies, KDM4/JHDM3, KDM5/JARID1, JMJD6, KDM3/JHDM2, and JMJ-C domain-only (Fig. 1), which were consistent with the *JMJ* family classification in *Arabidopsis* and rice. JMJD6 subfamily has two members in the Arabidopsis, rice, tomato, banana and melon (Fig. 1), and KDM4/JHDM3, KDM5/JARID1, and KDM3/JHDM2 subfamilies showed the most protein member among the five plants, indicating the gene conservation of the subfamilies in different species.

Gene duplication events within the genome promote the generation of new genes, and gene family expansion or contraction ensure their essential function in life activities. In melon, two genes (*CmJMJ3a* and *CmJMJ3b*) displayed a syntenic relationship, suggesting one gene duplication with no tandem duplication events for gene family expansion. The collinearity analyses showed that melon had a better genomic collinearity with cucumber than Arabidopsis, which was due to the nearer evolutionary relationship among *Cucurbitaceae* plants. The coding sequences of 17 *CmJMJ* genes had no premature stop codons, meaning that these genes encode functional proteins. *CmJMJ14* is located in chromosome 8, the remaining 16 genes are evenly distributed on chromosomes 1, 2, 3, 4, 5, 6, 9, and 12. All JMJ-C family members contain JMJ-C conserved domain, but this domain does not act alone in the histone demethylation process. Many conserved domains in JMJ-C proteins have DNA-binding functions, such as zf-4CXXC_R1, ARID, PLU-1, Zf-C5HC2, FYRN, and FYRC, and these DNA-binding domains contribute to the specific function of JMJ-C proteins [50]. The FYRN and FYRC conserved domains interact with the CTTGNNNNNNCAAG sequence of transcription factors *NAC050* and *NAC052* in *Arabidopsis* [51]. AtJMJ14 modulates plant defense against pathogens by regulating salicylic acid- and pipecolic acid-mediated defense pathway genes [52]. CmJMJ4, the homolog of AtJMJ14, had the same conserved FYRN and FYRC domains and salicylic acid response elements in gene sequence, which supposed to follow a similar gene regulation mechanism. The plant specific domains and motifs among the five CmJMJ subfamilies underlie their different activities in regulating biological processes.

Cis-acting elements in the gene promoter region are involved in the regulation of gene expression. We analyzed the promoters [53] of *CmJMJ* genes and found many cis elements associated with plant hormones, abiotic stress, and plant growth and development. Light-responsive elements were found in the promoter of all 17 *CmJMJs*. Auxin-responsive elements, abscisic acid- responsive elements, gibberellin-responsive elements, and MeJA-responsive elements were found in more than ten *CmJMJ* genes, respectively. Notably, the duplication genes *CmJMJ3a* and *CmJMJ3b* had common drought stress-responsive elements and hormone cis-elements, implying that they might be co-induced under the hormones and drought stresses. The hormone signaling pathways regulate the fruit development by activating their downstream genes. For example, Auxin/IAA may operate synergistically with ethylene in peach fruit by using *PpERF4* as a signaling component through auxin response factors *PpIAA1* and promote ABA biosynthesis through binding to and activation of the *PpNCED2* and *PpNCED3* promoters [54]. Cis-elements analysis indicated that the expression levels of the *CmJMJ* genes may be modulated by diverse environmental factors, such as light, hormones and abiotic stress.

We characterized the expression profile of the 17 *CmJMJ* genes by RNA-seq transcriptome data analyses and further investigated by RT-qPCR. Several *CmJMJ* genes were significantly expressed in both female and male flowers, which may participate in the regulation of melon floral organ development. The expression levels of four genes, *CmJMJ5a*, *CmJMJ9*, *CmJMJ5b* and *CmJMJ10*, showed a significant change during different fruit developmental stages, suggesting their potential role in fruit development and ripening. In tomato, knockdown of the *SlDML2* gene resulted in delayed fruit maturation and decreased 5-methylcytosine (5mC) DNA methylation levels at *CNR* locus [55, 56]. Tomato *SlJMJ6* and *SlJMJ7* genes were upregulated in early fruit development and downregulated in late fruit ripening [25, 42]. In banana (*Musa acuminata*), *MaJMJ* genes also showed specific expression during fruit development [43]. The correlated expression pattern of *JMJs* transcripts in the reproductive organs suggested their indispensable role in regulating fruit development in different plants. In conclusion, we identified 17 *JMJ-C* genes from *Cucumis melo* and characterized their sequence features, the protein characteristics, phylogenetic relationships, chromosomal localization, collinear synteny, and expression pattern analyses of *CmJMJ* genes, which will lay the foundation for further exploring their molecular functions.

## Materials and Methods

### Plant materials

The melon (*Cucumis melo* cv. Hetao) inbred line used in this study was cultivated in Dengkou County, Inner Mongolia. We have obtained permission to collect melon in Dengkou County. The original plants and planting materials were provided and authorized by Prof Hasi and his team. The Hetao melons were cultivated in the conventional cultivation method and bear one fruit per plant at the 3 or 4 sub-secondary node. Roots, stems, leaves, female and bisexual flowers and ovary at the day of anthesis were sampled from 60-day-old seedlings. The fruit pulp samples were collected 18 days (G stage) and 36 days (R stage) after pollination, as well as at the climacteric stage determined by a breathalyzer (C stage) and 48 h after the climacteric stage (P stage). All tissues were immediately frozen in liquid nitrogen and stored at -80 °C for quantitative real-time PCR.

### Identification and analysis of physicochemical properties of *CmJMJ*

The HMM (Hidden Markov Model) files of the JumonjiC (JMJ-C) domain (PF02373) from the Pfam protein family database (http://pfam.xfam.org/) [57] were employed as the seed model to search predicted proteins of JMJ-C from the melon genome database (http://cucurbitgenomics.org/), with the default setting. All *Arabidopsis* JMJ-C protein sequences were used as query sequence to preform Basic Local Alignment Search to search the melon JMJ-C family members, with an *E*-value of 1*e*^–5^. After filtering redundant sequences from the HMM and the protein blast outcomes, all retrieved sequences obtained by the combinatorial method were examined with the NCBI Conserved Domain Database (CDD) (https://www.ncbi.nlm.nih.gov/Structure/cdd/wrpsb.cgi) [58]. InterPro database (http://www.ebi.ac.uk/interpro/) [59] was used for accurate verification conserved protein domain (PF02373). The confirmed *CmJMJ* genes were named based on homology between the melon and *Arabidopsis* genes*.* The protein signatures, such as amino acids number (AAs), isoelectric point (pI) and molecular weight (Mw) of the CmJMJ proteins were acquired using prot-Param in ExPASy (https://web.expasy.org/protparam/) [60]. The subcellular localization of *CmJMJ* genes was predicted with the Plant-mPLoe server (http://www.csbio.sjtu.edu.cn/bioinf/Cell-PLoc-2/).

### Classification and Phylogenetic Analysis of* CmJMJ*

MUSCLE was used to perform multiple sequence alignments of the full-length amino acid sequences of all predicted melon (*Cucumis melo*) JMJ-C proteins and the orthologs. JMJ-C proteins sequences from tomato (*Solanum lycopersicum*), grape (*Vitis vinifera*), watermelon (*Citrullus lanatus*), and *Arabidopsis* (*Arabidopsis thaliana*) were acquired. The protein data of 21 AtJMJ were extracted from the TAIR database (https://www.arabidopsis.org), and the protein data of other three species were downloaded from Phytozome database (http://www.phytozome.net/eucalyptus). The phylogenetic tree was constructed by the Neighbor Joining (NJ) method [61] in MEGA7.0 software [62], with 1000 bootstrap replicates.

### Gene structure and conserved motif analysis of *CmJMJ*

The gene exon–intron structure information of the melon *JMJ-C* gene family was extracted from the melon genome database (http://cucurbitgenomics.org/) and visualized using the Redraw Gene Structure (From GFF/GTF3 File) function in TBtools. The conserved motifs and domains of the CmJMJ proteins were analyzed by the online software MEME (http://meme-suite.org/tools/meme) and TBtool software, respectively [63]. The visualized map of the motifs and conserved structural domains was performed by TBtool software.

### Chromosome location and collinearity analysis of *CmJMJ*

The chromosomal locations information of the *CmJMJs* were acquired from annotated gff3 files in the melon genome database (DHL92) and then analyzed by TBtools software. MCScanX with the default parameters was used to identify gene duplication events of *CmJMJs* in melon and their synchronous relationship with the homologous *JMJs* in Arabidopsis and cucumber. All the above results were drawn by the TBtool software.

### Cis-regulatory element analysis of* CmJMJ*

The upstream sequences of *CmJMJs* (defined as 2 kb away from the transcription start site) were downloaded and submitted to PlantCARE (http://bioinformatics.psb.ugent.be/webtools/plantcare/html/) [64] for the promoter cis-element analysis, and visualization was performed using TBtool software.

### Transcriptomic resources

To explore the expression pattern of the *CmJMJs*, the transcriptomic data of ‘Hetao’ melon *JMJ-C* genes in different tissues (roots, stems, leaves, flowers, male flowers, and ovaries) were used the NCBI sequence read archive (SRA) with accession number PRJNA803327. The RNA-seq data for melon different fruit development stages, including the growing stage (G stage), ripening stage (R stage), climacteric stage (C stage) and post climacteric stage (P stage), were downloaded from the NCBI sequence read archive (SRA) with accession numbers PRJNA543288. The heatmap was constructed from the FPKM normalized log-transformed values of the different samples and visualized via TBtool software.

### RNA extraction and quantitative RT-PCR

Different tissue samples of melon were extracted, and 1 μg of RNA was extracted from each sample. First-strand cDNA was synthesized by the PrimeScript® RT reagent Kit with gDNA Eraser according to the manufacturer’s instructions. The obtained cDNA was diluted tenfold as the template for quantitative real-time PCR, and *CmGAPDH* was used as the internal reference gene. The *CmJMJ* gene-specific primers were designed by Primer BLAST of NCBI (https://www.ncbi.nlm.nih.gov/tools/primer-blast/). RT-qPCR was performed using a 96-well Chromo4 Real-Time PCR system with SYBR® Premix Ex Taq™II (Takara RR820A). The RT-q PCR conditions were as follows: predenaturation at 95 °C for 30 s, followed by 40 cycles at 60 °C for 30 s. The relative gene expression values of *CmJMJ* were calculated with the 2^−ΔΔCt^ method. Three independent biological replicates and three technical replicates were used for each sample. The data analyses with the mean ± standard deviation of three replications were visualized in GraphPad Prism 8.

### Subcellular localization analysis of CmJMJ5a

To determine the subcellular localization pattern of CmJMJ5a, the coding sequence without the stop codon was amplified and inserted into the pCAMBIA1300-GFP vector with *XmaI* I and *BamH* I restriction enzymes to form the construct 35S:CmJMJ5a-GFP. The fusion construct (35S:CmJMJ5a-GFP) and control vector (35S:GFP) were transformed into *Agrobacterium tumefaciens* GV3101. The fusion expression vectors were transformed into tobacco (*N. benthamiana*) leaves for agroinfiltration as described previously [65]. The GFP fluorescence was visualized by a Nikon AX/AX R Confocal Microscope System. DAPI (40-6-diamidino-2-phenylindole) was used to stain the nuclei.

## Supplementary Information

**Additional file 1: Table S1. **Primer sequence and application

## Data Availability

Raw reads for RNA-Seq were downloaded from the NCBI Sequence Read Archive (SRA) database (https://www.ncbi.nlm.nih.gov/sra) under accession number PRJNA803327 (https://www.ebi.ac.uk/ena/browser/view/PRJNA803327) and PRJNA543288 (https://www.ebi.ac.uk/ena/browser/view/PRJNA543288). The datasets are available from the corresponding author on reasonable request.
