# Correction: Genome-wide identification and expression analysis of the JMJ-C gene family in melon (*Cucumis melo* L.) reveals their potential role in fruit development

**DOI:** 10.1186/s12864-025-11704-9

**Published:** 2025-05-26

**Authors:** Wuyun Jin, Wei Yan, Ming Ma, Agula Hasi, Gen Che

**Affiliations:** Key Laboratory of Herbage & Endemic Crop Biology, Ministry of Education, School of Life Sciences, Inner Mongolia University, Hohhot, 010070 China

**Correction**: ***BMC Genomics*** **24**, **771 (2023)**

10.1186/s12864-023-09868-3

Following publication of the original article, it was reported that there were incorrect values in the ‘CDS length/bp’ column and ‘AA’ column of Table 1.

The original Table 1 and the updated Table 1 with the corrected values highlighted in bold are given below.

Additionally, in the sub-section ‘Identification of *JMJC* genes in *Cucumis melo* L.’ the following sentence has been corrected (changes in bold):

- **Original sentence**: The protein length of CmJMJ showed various differences, which varied from 272 aa (*CmJMJ7*) to 1144 aa (CmJMJ6); the corresponding coding sequence length of the CmJMJ genes varied from 1242 bp (*CmJMJ7*) to 5454 bp (*CmJMJ6*); the molecular weights ranged from 46.68 kDa (*CmJMJ7*) to 208.73 kDa (*CmJMJ6*), and the predicted pI was between a minimum of 4.33 (*CmJMJ8*) and a maximum of 8.91 (*CmJMJ10*) (Table 1).
- **Corrected sentence**: The protein length of CmJMJ showed various differences, which varied from **413** aa (*CmJMJ7*) to **1817** aa (CmJMJ6); the corresponding coding sequence length of the CmJMJ genes varied from 1242 bp (*CmJMJ7*) to 5454 bp (*CmJMJ6*); the molecular weights ranged from 46.68 kDa (*CmJMJ7*) to 208.73 kDa (*CmJMJ6*), and the predicted pI was between a minimum of 4.33 (*CmJMJ8*) and a maximum of 8.91 (*CmJMJ10*) (Table 1).

The original article has been updated.

**Original Table ****1**.

**Table 1:** Chromosomal location of melon *CmJMJs* and physicochemical properties of proteins

Gene name	Gene ID	Chromosome	Location	CDS length/bp	Protein			
					AA	Mw (kDa)	pI	Subcellular localization
*CmJMJ1*	MELO3C019813.2	3	23,489,140–23,497,463 (-)	4848	1098	180.47	6.74	Nuclear
*CmJMJ2*	MELO3C002327.2	12	24,822,711–24,830,280 (-)	2562	581	93.82	6.22	Nuclear
*CmJMJ3a*	MELO3C003435.2	4	1,165,795–1,173,643 ( +)	1923	428	72.92	7.97	Nuclear
*CmJMJ3b*	MELO3C013826.2	6	33,793,389–33,804,248 (-)	2679	606	100.43	7.69	Nuclear
*CmJMJ4*	MELO3C021809.2	12	17,928,201–17,933,964 ( +)	3081	660	116.30	5.86	Nuclear
*CmJMJ5a*	MELO3C008714.2	5	17,778,859–17,787,934 ( +)	3705	830	138.28	7.35	Nuclear
*CmJMJ5b*	MELO3C012748.2	1	24,084,739–24,089,250 ( +)	2493	542	93.72	6.63	Nuclear
*CmJMJ6*	MELO3C005670.2	9	23,335,384–23,355,501 (-)	5454	1144	208.73	6.82	Nuclear
*CmJMJ7*	MELO3C015209.2	2	5,984,904–5,989,035 ( +)	1242	272	46.68	5	Nuclear
*CmJMJ8*	MELO3C026280.2	2	26,273,023–26,277,717 (-)	1365	296	52.18	4.33	Nuclear
*CmJMJ9*	MELO3C011038.2	3	29,182,217–29,192,951 (-)	2910	622	111.01	5.12	Nuclear
*CmJMJ10*	MELO3C009577.2	4	31,073,510–31,075,890 ( +)	1539	337	62.98	8.91	Nuclear
*CmJMJ11*	MELO3C004232.2	5	25,411,787–25,417,256 ( +)	1989	442	103.88	7.13	Nuclear
*CmJMJ12*	MELO3C025053.2	9	15,494,523–15,505,749 ( +)	3075	697	116.20	6.91	Nuclear
*CmJMJ13*	MELO3C006019.2	6	605,711–619,023 ( +)	2946	682	111.29	7.65	Nuclear
*CmJMJ14*	MELO3C007332.2	8	2,239,225–2,245,012 (-)	3018	659	114.29	7.77	Nuclear
*CmJMJ15*	MELO3C015807.2	1	30,446,344–30,468,380 (-)	1797	387	67.49	5.99	Nuclear

**Corrected Table ****1**.

**Table 1:** Chromosomal location of melon CmJMJs and physicochemical properties of proteins

Gene name	Gene ID	Chromosome	Location	CDS length/bp	Protein			
					AA	Mw (kDa)	pI	Subcellular localization
*CmJMJ1*	MELO3C019813.2	3	23,489,140 − 23,497,463 (-)	4848	**1615**	180.47	6.74	Nuclear
*CmJMJ2*	MELO3C002327.2	12	24,822,711 − 24,830,280 (-)	2562	**853**	93.82	6.22	Nuclear
*CmJMJ3a*	MELO3C003435.2	4	1,165,795-1,173,643 (+)	1923	**640**	72.92	7.97	Nuclear
*CmJMJ3b*	MELO3C013826.2	6	33,793,389 − 33,804,248 (-)	2679	**892**	100.43	7.69	Nuclear
*CmJMJ4*	MELO3C021809.2	12	17,928,201 − 17,933,964 (+)	3081	**1026**	116.30	5.86	Nuclear
*CmJMJ5a*	MELO3C008714.2	5	17,778,859 − 17,787,934 (+)	3705	**1234**	138.28	7.35	Nuclear
*CmJMJ5b*	MELO3C012748.2	1	24,084,739 − 24,089,250 (+)	2493	**830**	93.72	6.63	Nuclear
*CmJMJ6*	MELO3C005670.2	9	23,335,384 − 23,355,501 (-)	5454	**1817**	208.73	6.82	Nuclear
*CmJMJ7*	MELO3C015209.2	2	5,984,904-5,989,035 (+)	1242	**413**	46.68	5	Nuclear
*CmJMJ8*	MELO3C026280.2	2	26,273,023–26,277,717 (-)	1365	**454**	52.18	4.33	Nuclear
*CmJMJ9*	MELO3C011038.2	3	29,182,217 − 29,192,951 (-)	2910	**969**	111.01	5.12	Nuclear
*CmJMJ10*	MELO3C009577.2	4	31,073,510 − 31,075,890 (+)	**1662**	**553**	62.98	8.91	Nuclear
*CmJMJ11*	MELO3C004232.2	5	25,411,787 − 25,417,256 (+)	**2730**	**909**	103.88	7.13	Nuclear
*CmJMJ12*	MELO3C025053.2	9	15,494,523 − 15,505,749 (+)	3075	**1024**	116.20	6.91	Nuclear
*CmJMJ13*	MELO3C006019.2	6	605,711 − 619,023 (+)	2946	**981**	111.29	7.65	Nuclear
*CmJMJ14*	MELO3C007332.2	8	2,239,225-2,245,012 (-)	3018	**1005**	114.29	7.77	Nuclear
*CmJMJ15*	MELO3C015807.2	1	30,446,344 − 30,468,380 (-)	1797	**598**	67.49	5.99	Nuclear

